# Granulocytic Sarcoma by AML M4eo (inv16) after Allogeneic Stem Cell Transplantation without Bone Marrow Involvement

**DOI:** 10.1155/2011/692982

**Published:** 2011-09-05

**Authors:** Stephan Zaenker, Stefan Schweyer, Justin Hasenkamp, Lorenz Truemper, Gerald Wulf

**Affiliations:** ^1^Department of Haematology/Oncology, University Medicine Goettingen, Robert-Koch-Street 40, 37075 Goettingen, Germany; ^2^Center of Pathology, University Medicine Goettingen, Robert-Koch-Street 40, 37075 Goettingen, Germany

## Abstract

Granulocytic sarcoma (GS) represents a rare type of extramedullar manifestation from the acute myeloid leukaemia (AML). We report the case of a patient with recurrences of AML M4eo leukaemia in the uterus and the small intestine at 3 and 5 years, respectively, after matched related peripheral blood stem cell transplantation (PBSCT). The patient underwent the withdrawal of immunosuppression, hysterectomy, and local irradiation at first relapse, as well as systemic chemotherapy and donor lymphocyte infusions at second recurrence, inducing a second and third complete remission, respectively. At year six after transplantation, the patient experienced disease progression by meningeosis leukaemia to which she succumbed despite intrathecal chemotherapy. Following allogeneic stem cell transplantation, awareness for atypical manifestations of granulocytic sarcoma appears prudent, the cellular immunotherapy should aim at immunological disease control.

## 1. Introduction

Granulocytic sarcoma is rare extramedullar manifestations of the AML, occurring particularly in patients with AML FAB M4/M5 morphology, AML t(8;21) or inv(16), as well as leukemias with CD13- and CD14-positive immunophenotype or high initial blast counts in the peripheral blood [1–4]. GS mostly involves the skin, lymph nodes, and soft tissue, furthermore, in rare cases manifestations in visceral organs have also been reported. Of those, disease manifestations in the ovary or the stomach have been reported particularly in patients with eosinophilic differentiation (M4eo) and inversion 16 [4–11]. GS characteristically expresss CD68 as the most common marker followed by myeloperoxidase (MPO) [1].

## 2. Case Report

In the case of the 51-year old proposita, the diagnosis of an AML M4eo was confirmed morphologically and cytogenetically by the characteristic inversion (16) (p13q22) in 12/2003. Because of a punctio sicca, no blood from the bone marrow could be aspirated for FACS analysis. A complete remission, also on cytogenetic level, was achieved after the first cycle of cytosine arabinoside, idarubicin, and etoposide induction therapy. This outcome was consolidated by high-dose chemotherapy with cytosine arabinoside and mitoxantrone as well as consecutive allogeneic PBSCT from an HLA-matched sibling donor after total body irradiation/cyclophosphamide (TBI/Cy) in 05/2004. Four weeks after the PBSCT, a 100% donor chimerism was detected. The patient experienced chronic graft versus host disease (GvHD), necessitating a low-dose systemic steroid treatment.

**Figure 1:**
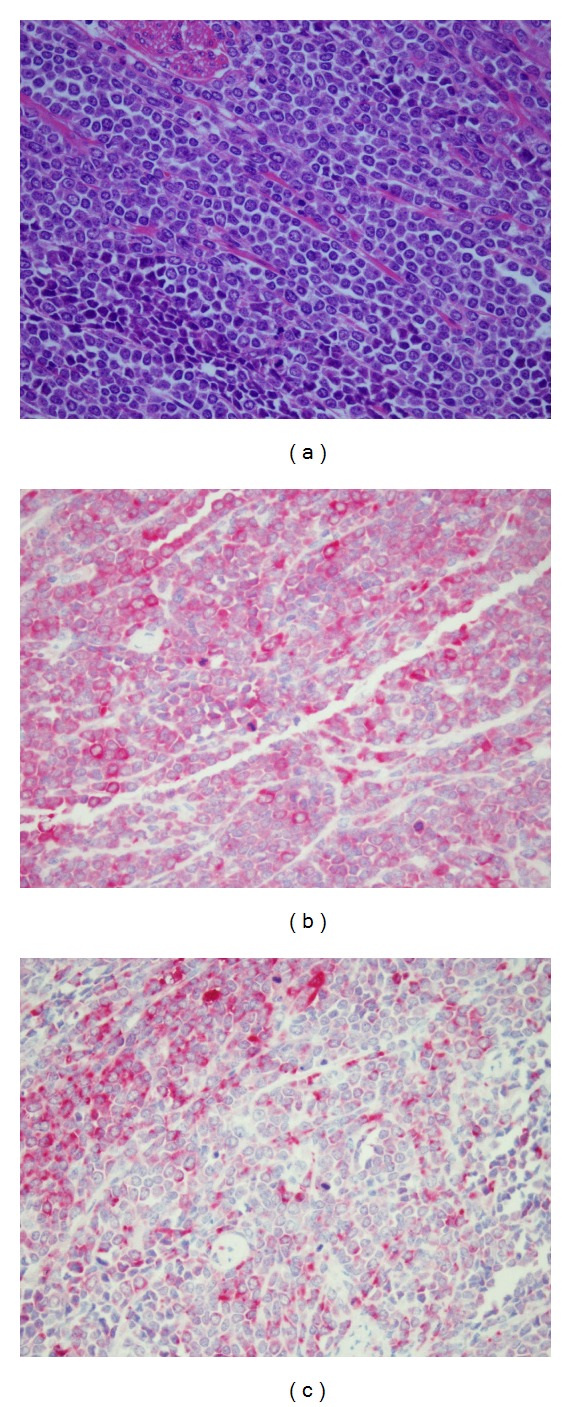
Immunohistology of granulocytic sarcoma. Tumor formation by monomorphic blast cells ((a), HE-stain). Significant proportions of the blast cells were positive for myeloperoxidase (b) and the monocyte marker CD68 (c).

 In 11/2007, a hysterectomy was performed after the occurrence of a painful uterine tumor, yielding the diagnosis of a GS-expressing MPO, the monocyte marker CD68 and KiM1P, without any bone marrow manifestation of the AML (Figure 1). The donor chimerism remains 100%. The surgery was followed by radiotherapy of the pelvis and withdrawal of immunosuppressive medication, leading to limited GvHD with cutaneous and mucocutaneous manifestations.

 In 11/2008, recurrent epigastric pain leads to a gastroscopy documenting again a GS (expression of CD68 and MPO) of the duodenum, without bone marrow infiltration and detectable inversion 16 and still with 100% donor chimerism. The patient received high-dose salvage chemotherapy with cytosine arabinoside and mitoxantrone, followed by donor lymphocyte infusions (DLI) inducing a third complete remission. However, in 04/2009 a meningeosis leukaemia (227 cells/mm^3^ liquor, expression of CD68 and MPO) was diagnosed, and treatment with intrathecal cytosine arabinoside initiated. The patient received no systemic chemotherapy because of a reduced general state of health and died from meningeal leukemia progression in 03/2010.

## 3. Discussion

Granulocytic sarcoma is rare manifestation of the AML and associated with diverse cytogenetic changes in the leukemic blast, in particular the core binding factor leukemia AML M2 t(8;21) [1–3, 12]. Very rarely observed cases of GS originating from blast cells with inv (16) have also been reported, predominantly occurring with intestinal manifestation, similar to the findings in the proposita [4, 7, 11, 12]. The prognosis of patients with granulocytic sarcoma is generally dismal especially in the AML relapse situation. In a retrospective investigation of 24 patients with GS overall survival at five years amounted to an average of only 21% [13]. Systemic chemotherapy similar to the AML patterns are offered to most of the patients, whereas the role of primary or consolidating local irradiation is currently not clear [13]. For the rare cases of AML disease relapse manifesting as GS, the standard of care yet remains to be defined.

Immunological control, that is exploiting the graft-versus-leukeamia (GvL) effect after allogeneic stem cell transplantation, appears as a promising approach in such patients. The relapse situation of the proposita, that is, the recurrent visceral organ manifestations without bone marrow involvement suggests that immunological control protected the bone marrow but was not sufficient to eradicate the disease at organ sites with immunological privileges. Interestingly, Maeng et al. [6] also reported about a patient with an isolated extramedullary relapse of AML in the uterus, occurring two years after allogeneic PBSCT. The course of disease reported here might show that patients with GS may benefit from combination of local control, chemotherapy, and exploiting the GvL effect. We conclude that awareness of disease manifestations at immunologically privileged sites appears prudent, and that optimal management of patients with recurring GS remains to be defined.

## References

[1] Pileri SA, Ascani S, Cox MC (2007). Myeloid sarcoma: clinico-pathologic, phenotypic and cytogenetic analysis of 92 adult patients. *Leukemia*.

[2] Paydas S, Zorludemir S, Ergin M (2006). Granulocytic sarcoma: 32 cases and review of the literature. *Leukemia and Lymphoma*.

[3] Byrd JC, Edenfield JW, Shields DJ, Dawson NA (1995). Extramedullary myeloid cell tumours in acute nonlymphocytic leukaemia: a clinical review. *Journal of Clinical Oncology*.

[4] Zhang XH, Zhang R, Li Y (2010). Granulocytic sarcoma of abdomen in acute myeloid leukemia patient with inv(16) and t(6;17) abnormal chromosome: case report and review of literature. *Leukemia Research*.

[5] Pathak B, Bruchim I, Brisson ML, Hammouda W, Bloom C, Gotlieb WH (2005). Granulocytic sarcoma presenting as tumors of the cervix. *Gynecologic Oncology*.

[6] Maeng H, Cheong JW, Lee ST (2004). Isolated extramedullary relapse of acute myelogenous leukemia as a uterine granulocytic sarcoma in an allogeneic hematopoietic stem cell transplantation recipient. *Yonsei Medical Journal*.

[7] Kohl SK, Aoun P (2006). Granulocytic sarcoma of the small intestine. *Archives of Pathology and Laboratory Medicine*.

[8] Drinkard LC, Waggoner S, Stein RN, Byrne RA, Larson RA (1995). Acute myelomonocytic leukemia with abnormal eosinophils presenting as an ovarian mass: a report of two cases and a review of the literature. *Gynecologic Oncology*.

[9] Yavuz S, Paydas S, Disel U, Erdogan S (2004). Ovarian granulocytic sarcoma. *Leukemia and Lymphoma*.

[10] Garcia MG, Deavers MT, Knoblock RJ (2006). Myeloid sarcoma involving the gynecologic tract: a report of 11 cases and review of the literature. *American Journal of Clinical Pathology*.

[11] Choi ER, Ko YH, Kim SJ (2001). Gastric recurrence of extramedullary granulocytic sarcoma after allogneic stem cell transplantation for acute myeloid leukemia. *Journal of Clinical Oncology*.

[12] Schäfer HS, Becker H, Schmitt-Gräff A, Lübbert M (2008). Granulocytic sarcoma of core-binding factor (CBF) acute myeloid leukemia mimicking pancreatic cancer. *Leukemia Research*.

[13] Lan TY, Lin DT, Tien HF, Yang RS, Chen CY, Wu K (2009). Prognostic factors of treatment outcomes in patients with granulocytic sarcoma. *Acta Haematologica*.

